# The effect of traumatic brain injury and post‐traumatic stress disorders on AD pathologies, small vessel changes and ultimately cognitive function

**DOI:** 10.1002/alz.093215

**Published:** 2025-01-09

**Authors:** Francesca Sibilia, Steven Cen, Jeiran Choupan, Saman Hazany, Rene Ayala, Jocelyn Cheng, Tiana Nejatpoor, Nasim Sheikh‐Bahaei

**Affiliations:** ^1^ Stevens Neuroimaging and Informatics Institute, Keck School of Medicine, University of Southern California, Los Angeles, CA USA; ^2^ Keck School of Medicine, University of Southern California, Los Angeles, CA USA; ^3^ USC Mark and Mary Stevens Neuroimaging and Informatics Institute, Keck School of Medicine, University of Southern California, Los Angeles, CA USA; ^4^ Keck School of Medicine of USC, LA, CA USA; ^5^ Keck school of Medicine of USC, LA, CA USA; ^6^ University of Southern California, LA, CA USA

## Abstract

**Background:**

Although many studies have shown that traumatic brain injury (TBI) and post‐traumatic stress disorder (PTSD) regardless of their severity, are associated with a significantly increased risk of all‐cause dementia, the specific pathophysiological mechanisms that underlie these associations remain poorly understood, resulting in discordance findings among different studies and contradictory claims in the current literature.

In this study we investigated the effect of TBI and PTSD on the level of amyloid, tau, as well as markers of small vessel health including white matter hyperintensity and perivascular spaces and consequently assessed their effect on the cognitive function in order to understand the pathways through which TBI and PTSD may result in dementia.

**Method:**

The participants in this study were MCI cases drawn from the ADNI‐DOD (n=58). Cognitive performance was assessed using the Mini‐Mental State Exam (MMSE) and Clinical Dementia Rating (CDR). Inclusion criteria for TBI involved the history of moderate to severe nonpenetrating head trauma as well as combat history or presence in the battlefield. Inclusion in the PTSD group required a minimum Clinician Administered PTSD Scale (CAPS) score of 40 points. PET images with tracers AV1451 and AV45 were obtained. Perivascular spaces (PVS) volume fraction in the total white matter and basal ganglia was obtained using automated segmentation pipeline developed at our institute, as well as the grade of white matter hyperintensities.

**Result:**

The volume of PVS in the white matter was found to be significantly higher in veterans with PTSD (p=0.01) or with TBI (p=0.02) after age adjustment. Elevated amyloid was also found in veterans with PTSD 1.3 (1.23‐1.39) vs. 1.23 (1.1‐1.53), p=0.05 and trend of increased amyloid with TBI 1.3 (1.18‐1.44) vs. 1.28 (1.23‐1.31), p=0.09. Our day also indicates a strong correlation between amyloid and CDR (adjusted ranking score correlation of 0.45 (p<0.01) among veteran sample.

**Conclusion:**

Preliminary evidence showed the association from PTSD to amyloid as well as TBI to amyloid. Since amyloid strongly associated with cognitive function, it may suggest a biological pathway from PTSD and/or TBI through amyloid deposition to cognitive decline in veteran. Larger sample will be needed to confirm the causal pathway.

